# Adipose Tissue Macrophage Polarization in Healthy and Unhealthy Obesity

**DOI:** 10.3389/fnut.2021.625331

**Published:** 2021-02-17

**Authors:** Alistaire D. Ruggiero, Chia-Chi Chuang Key, Kylie Kavanagh

**Affiliations:** ^1^Section on Comparative Medicine, Department of Pathology, Wake Forest University School of Medicine, Winston-Salem, NC, United States; ^2^Section on Molecular Medicine, Department of Internal Medicine, Wake Forest University School of Medicine, Winston-Salem, NC, United States; ^3^Department of Biomedicine, University of Tasmania, Hobart, TAS, Australia

**Keywords:** adipose, macrophage, metabolically healthy, metabolically unhealthy, obesity

## Abstract

Over 650 million adults are obese (body mass index ≥ 30 kg/m^2^) worldwide. Obesity is commonly associated with several comorbidities, including cardiovascular disease and type II diabetes. However, compiled estimates suggest that from 5 to 40% of obese individuals do not experience metabolic or cardiovascular complications. The existence of the metabolically unhealthy obese (MUO) and the metabolically healthy obese (MHO) phenotypes suggests that underlying differences exist in both tissues and overall systemic function. Macrophage accumulation in white adipose tissue (AT) in obesity is typically associated with insulin resistance. However, as plastic cells, macrophages respond to stimuli in their microenvironments, altering their polarization between pro- and anti-inflammatory phenotypes, depending on the state of their surroundings. The dichotomous nature of MHO and MUO clinical phenotypes suggests that differences in white AT function dictate local inflammatory responses by driving changes in macrophage subtypes. As obesity requires extensive AT expansion, we posit that remodeling capacity with adipose expansion potentiates favorable macrophage profiles in MHO as compared with MUO individuals. In this review, we discuss how differences in adipogenesis, AT extracellular matrix deposition and breakdown, and AT angiogenesis perpetuate altered AT macrophage profiles in MUO compared with MHO. We discuss how non-autonomous effects of remote organ systems, including the liver, gastrointestinal tract, and cardiovascular system, interact with white adipose favorably in MHO. Preferential AT macrophage profiles in MHO stem from sustained AT function and improved overall fitness and systemic health.

## Introduction

As of February 2020, more than 1.9 billion adults worldwide were overweight [body mass index (BMI): 25–29.9 kg/m^2^], and over 650 million were obese (BMI ≥ 30 kg/m^2^) (1). Obesity decreases lifespan and increases the risk of developing hypertension, dyslipidemia, and type II diabetes (T2D) (2–4). Despite the number and variety of deployed weight-loss interventions, very few overweight or obese patients maintain weight loss over time, and globally, the number of obese individuals continues to increase (5). Across the BMI spectrum, not all obese individuals suffer the same comorbidities. Roughly 60% of obese individuals present with dysglycemia, hypertension, and/or dyslipidemia, and cutoff criteria associated with each of these maladies define obesity as either healthy [metabolically healthy obese (MHO)] or unhealthy [metabolically unhealthy obese (MUO)] (6, 7). The majority of individuals are classified as MUO; however, between 5 and 40% of obese individuals do not present with metabolic abnormalities and are defined as MHO (6–8). Definitions of MHO vary, as some studies identify only insulin-sensitive individuals as MHO, whereas others identify individuals with two or fewer metabolic abnormalities as MHO (7, 9, 10). A recently proposed definition of MHO identifies individuals based on the diagnosis of obesity and the following criteria: serum triglycerides ≤150 mg/dl, HDL-cholesterol concentrations >40 mg/dl in men or >50 mg/dl in women, systolic blood pressure ≤130 mmHg, diastolic blood pressure ≤85 mmHg, no antihypertensive treatment as an alternative indicator, fasting blood glucose ≤100 mg/dl, and no treatment with glucose lowering agents (11). Significant controversy exists over the definitions and stability of MHO classifications. There is no universally accepted definition of MHO; many MHO individuals progress to MUO over time, and, although MHO individuals do have higher all-cause mortality and an increased risk of cardiovascular events compared with healthy lean individuals (12, 13), they are at a decreased risk of cardiovascular complications and all-cause mortality compared with the MUO individual. Despite this controversy, understanding the biological mechanisms that maintain metabolic health with overt obesity would aid the development of therapeutics to convert MUO individuals to MHO and ultimately reduce the financial burden of obesity-related comorbidities.

**Figure 1:**
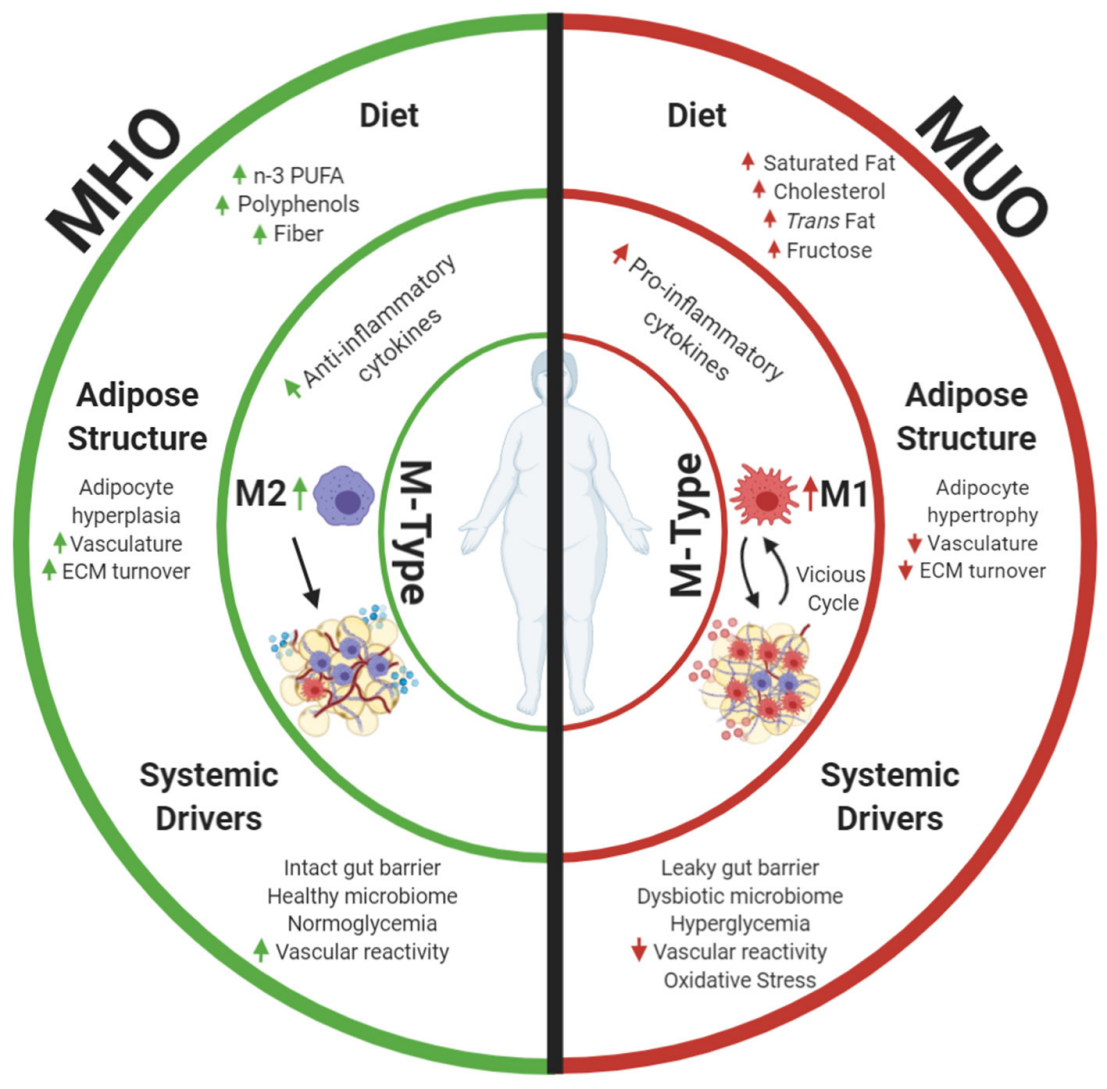
Metabolically healthy obesity (MHO) and metabolically unhealthy obesity (MUO) may be defined by differences in AT. This review focuses on macrophage phenotypes (M-Types) as a key element driving adipose health, as these immune cells have potent effects on the local AT niche and are influenced by diet and other systemic health characteristics. Differences in dietary components and aspects of adipose expansion perpetuate MHO and MUO adipose macrophage phenotypes. Increased consumption of omega 3 polyunsaturated fatty acids (n-3 PUFAs), polyphenols, and fiber results in anti-inflammatory adipose macrophage (M2) programming in MHO. These dietary components combined with improved white adipose adipogenesis and corresponding adipocyte hyperplasia increase tissue vascularization and extracellular matrix (ECM) turnover, and downstream anti-inflammatory signaling propagates anti-inflammatory M2 macrophage proliferation while abating harmful pro-inflammatory M1 macrophage recruitment into tissue. Parallel to effects of diet and adipose structure, systemic drivers, including functional liver-adipose cross talk and improved vasoreactivity, also decrease MHO AT pro-inflammatory signaling and maintain insulin sensitivity. Alternatively, increased consumption of saturated fat, cholesterols, *trans* fat, and fructose incites pro-inflammatory macrophage recruitment in MUO adipose. Consumption of these dietary components in conjunction with dysfunctional adipogenesis results in adipocyte hypertrophy. This combined with decreased angiogenic signals, disrupted ECM turnover, and downstream pro-inflammatory cytokine secretion stimulates pro-inflammatory M1 macrophage recruitment. Systemic dysfunction in the form of a decreased vasoreactivity and oxidative stress concurrently fosters insulin resistance while promoting pro-inflammatory M1 macrophage recruitment into MUO adipose. Once M1 macrophages enter the tissue, they secrete additional pro-inflammatory cytokines that recruit more M1 macrophages. This vicious cycle of inflammation, perpetuation of unhealthy AT, and greater multisystemic dysfunction characterize MUO individuals who are defined by increased expression of metabolic syndrome components. Created with Biorender.com.

Although obesity results in white adipose tissue (AT) expansion, maintenance of metabolic function may underlie MHO individuals' superior metabolic homeostasis. AT functions as an endocrine organ that maintains energy equilibrium, but function can differ by location. White AT accumulates throughout the body, including in the epicardial, mesenteric, omental, retroperitoneal, gonadal, subcutaneous abdominal, gluteal, and femoral regions (14). Intra-abdominal, or visceral, and subcutaneous white AT depots perform different functions and thus differentially impact metabolic health. Visceral AT accumulation is positively associated with cardiometabolic risk factors (15) and correlation with decreased insulin sensitivity (16). On the contrary, subcutaneous white AT accumulation protects against cardiometabolic risk factors (15) and corresponds with maintained insulin sensitivity (17), as evidenced by subcutaneous adipose transplantation into visceral depots alleviating metabolic dysregulation (18). Both adipocytes and immune cells in AT express and secrete bioactive hormones and signaling proteins that regulate metabolism (19–21). The goal of this review is to summarize macrophages as key AT immune cells and their influences, which drive tissue function (Figure 1). Elucidating changes in white AT composition is crucial to unraveling the mechanisms behind the observed metabolic differences between MHO and MUO groups.

Obesity-associated AT expansion often results in the accumulation of immune cells, including macrophages, contributing to low-grade chronic inflammation. Macrophages are the most abundant leukocytes in AT and assist in regulating physiological processes, including tissue remodeling and insulin sensitivity. Macrophage accumulation was originally thought to be universally pro-inflammatory and contributory to insulin resistance. However, macrophage subtypes stimulate different responses within AT. Macrophage subtypes exist along a continuum, as they demonstrate variable metabolic activation and ranges in inflammatory signaling (22, 23). As such, they are often classified by whether they are more pro-inflammatory or more anti-inflammatory. M1 macrophages are thought to be more pro-inflammatory and secrete pro-inflammatory cytokines that ultimately inhibit proper insulin signaling in adipocytes (23, 24). Contrarily, M2 macrophages are thought to be more anti-inflammatory and secrete anti-inflammatory cytokines that maintain functional insulin signaling (23, 24).

In obese states, macrophages play crucial roles in damage response. Macrophage polarization patterns are influenced by environmental cues and inflammatory signaling (23), and macrophages accumulate when danger signals propagate and incite more inflammation to manage their resolution. In obese states, macrophages clear dead adipocytes and other cell debris, exocytose excess lipid, secrete both pro- and anti-inflammatory cytokines, and contribute to adipose remodeling (23). Macrophage responses are interrupted by insulin resistance, hypoxia, and reactive oxygen species generation, metabolic endotoxemia or cell senescence, and death. While macrophage accumulation in obese individuals is typically affiliated with inflammation and these downstream consequences, healthy obese individuals do not demonstrate the same levels of inflammation as their MUO counterparts. In this review, we discuss how differences in white AT components give rise to contrasting macrophage M-phenotypes in MHO compared with MUO persons. We also discuss how diet and aspects of systemic metabolic health impact AT macrophages.

## Adipose Macrophage Subtypes

Obesity alone incites the recruitment and proliferation of AT macrophages, the predominant adipose leukocyte population (25, 26). As plastic cells that respond to their microenvironments, macrophages range from highly pro-inflammatory, or M1-like, to highly anti-inflammatory, or M2-like (27). M1 macrophages fight against intracellular pathogens, are induced by pro-inflammatory factors including lipopolysaccharide and interferon-γ, and secrete inflammatory cytokines including interleukin (IL)-6, IL-1β, and monocyte chemoattractant protein-1 (MCP-1) (28). Hematopoietic-derived M1 macrophages utilize glycolysis and are recruited into AT and where they can proliferate (29–31). M2 macrophages, on the other hand, contribute to tissue repair and produce anti-inflammatory cytokines, including IL-4 and IL-13. Contrary to M1 macrophages, yolk sac-derived M2 macrophages utilize oxidative phosphorylation (31). White AT homeostasis requires a balance of both these pro- and anti-inflammatory macrophage subtypes. Excess M1 macrophage infiltration results in increased tissue inflammation, whereas an overabundance of M2 macrophages can lead to aberrant fibrogenesis, limiting the remodeling required for AT to respond to changing lipid storage needs (32, 33). In diet-induced obese states, macrophage polarization shifts from a 4:1 M2-to-M1 ratio in lean animals to a 1.2:1 ratio in obese animals, as M1 macrophages are recruited into white AT (29).

MHO individuals display an anti-inflammatory adipose macrophage profile that more closely resembles that of metabolically healthy lean individuals, including an increased M2:M1 ratio (34). Of note, MHO individuals likely accumulate metabolically active adipose macrophages, which is a subtype that may be distinct from coarsely defined M1 or M2. Although we will focus primarily on M1 and M2 macrophages in the remainder of this text, metabolically active macrophages are associated with decreased nicotinamide adenine dinucleotide phosphate oxidase 2, which reduces the inflammation associated with obesity and downstream insulin resistance (30, 35, 36). These macrophages are thought to better regulate lipid, catecholamine, and iron availability and perform other functions, including modulating local inflammation and clearing dead adipocytes during prolonged obesity (30, 35, 36). This suggests that, in contrast to MUO, MHO individuals' anti-inflammatory AT macrophage profile helps maintain insulin and glucose regulation and deters pro-inflammatory macrophage recruitment. In the following sections, we will discuss the relationships between aspects of AT function, macrophage polarization, and metabolic regulation in MHO compared with MUO.

## Adipocyte Function and Adipogenesis

MHO adipocyte function mediates anti-inflammatory AT macrophage activation and polarization (37). Adipocytes secrete cytokines, depending on their inflammatory state, that influence immune cells. Secretion of Th2 cytokines, including IL-4 and IL-13, induces macrophage peroxisome proliferator-activated receptor (PPAR)δ activation, a regulator of fatty acid metabolism (38), which also improves whole-body insulin sensitivity (39). Insulin-sensitive obese individuals have higher PPARγ (i.e., PPARγ2) messenger RNA (mRNA) expression levels in peripheral blood mononuclear cell and their visceral adipose than do insulin-resistant obese individuals (40, 41). Ablation of both PPARγ, a regulator of adipogenesis and lipogenesis, and PPARδ renders macrophages unable to transition to the M2 subtype (38, 42, 43). Therefore, in MHO individuals, adipocyte cytokine secretion and its downstream effects on fatty acid metabolism and adipogenesis regulators incite M2 macrophage polarization and whole-body insulin sensitivity while promoting sufficient adipogenesis (i.e., proliferation and differentiation of preadipocytes) to manage the concurring nutrient overload.

Fluctuations in AT macrophage ratios correspond to changes in preadipocyte differentiation and adipogenic signaling. The factors secreted by pro-inflammatory M1 macrophages possess anti-adipogenic properties (44). Unlike M1 macrophages, both M2 macrophages and inactive macrophages promote preadipocyte survival by releasing a platelet-derived growth factor (PDGF) (45). However, excess M2 macrophages have been shown to impair preadipocyte differentiation through the transforming growth factor-beta (TGF-β) pathway (46). The effects of AT macrophage balance on preadipocyte differentiation and adipose expansion require further investigation (47); however, differences in the AT macrophage profiles of MHO and MUO individuals likely drive the rate and format of adipogenesis, as *in vitro* differentiation protocols illustrate that adipogenesis is greater in MHO than MUO people (48–51). The nuclear hormone receptor PPARγ acts as a master transcription factor of adipocyte differentiation by inducing and maintaining the expression of key adipogenic genes, such as GLUT4 and adiponectin, which are necessary for normal adipocyte function and downstream insulin sensitivity (52). Diabetic patients treated with PPARγ-activating thiazolidinediones, a class of antidiabetic drugs, often experience weight gain in the form of subcutaneous AT expansion. Accordingly, the subcutaneous AT expansion that occurs through adipocyte hyperplasia (increasing number of cells through differentiation of new adipocytes) appears metabolically favorable and contributes to systemic insulin sensitization (53), and the same PPARγ signaling promotes an M2-positive AT balance. In obese states, AT macrophages provide signals that communicate with mature adipocytes and preadipocytes to incite either adipocyte hypertrophy or preadipocyte differentiation and adipocyte hyperplasia. In MHO, *de novo* adipogenesis driven by improved glucose uptake and anti-inflammatory signaling—including increased PPARγ and adiponectin—results in smaller, more numerous adipocytes (54); the resulting adipocyte hyperplasia maintains a more anti-inflammatory AT macrophage profile (54). In MUO, hypertrophic adipocytes communicate with recruited M1 macrophages and secrete pro-inflammatory leukotrienes, such as LTB4, which inhibit insulin signaling in metabolic tissues and thus further recruit more pro-inflammatory macrophages (55). Proteomic analyses of visceral AT from T2D MUO obese individuals reveal mitochondrial dysfunction and reduced adipocyte differentiation, which additionally incites M1 macrophage recruitment (56). This vicious cycle continues with tumor necrosis factor-alpha and IL-1β secreted by M1 macrophages, further impairing adipogenic differentiation, with reduced adipogenic gene expression in subcutaneous AT and, in some cases, increased numbers of small adipocytes (48, 57, 58). In contrast, normoglycemic obese individuals show an increase in the percent of adipose progenitors within their tissue compared with both pre-diabetic and T2D obese subjects (59). MHO individuals benefit from a positive insulin sensitizing cycle, where anti-inflammatory signals from adipocytes result in M2 macrophage polarization, which promotes healthy adipocyte hyperplasia and further anti-inflammatory signaling from adipocytes.

## Extracellular Matrix Remodeling

Healthy AT expansion requires extracellular matrix (ECM) remodeling. Adipocytes are surrounded by a network of ECM proteins that serve as a mechanical support and respond to different signaling events (60). AT expansion relies on adaptive cellular and extracellular responses to prevent ectopic lipid deposition and lipotoxicity (61–63). Within AT, collagens produced primarily by adipocytes and endothelial cells comprise most non-cell tissue mass, whereas integrins are the major tissue receptors for cell adhesion to ECM proteins (64, 65). Increased interstitial fibrosis due to excess collagen deposition likely decreases ECM flexibility and reduces tissue plasticity, which leads to adipocyte dysfunction and immune cell infiltration (66). Insulin-resistant individuals demonstrate aberrant ECM deposition and insufficient ECM breakdown (59, 67).

Important protein families that comprise the ECM include matrix metalloproteinases (MMPs), enzymes that process and degrade pericellular substrates and play a vital role in regulating ECM remodeling in normal and disease states (68). Tissue inhibitors of metalloproteinases (TIMPs), which comprise a family of four protease inhibitors, inhibit MMPs to achieve a balance in production and breakdown (69). Adipose expression of MMP-9, increased in MUO compared with MHO (38, 70), positively correlates with insulin resistance and cardiovascular risk in obese persons. Similarly, visceral AT MMP-14 expression correlates with adipose accumulation and insulin resistance in women (71). MMP-11 is also increased in the white adipose of obese insulin-resistant mice (72). TIMP-3 regulates white adipose inflammation and insulin sensitivity, and its deletion in mice increases M1 macrophage accumulation in white AT (73), whereas its overexpression in macrophages resulted in improved glucose tolerance and insulin sensitivity and decreased inflammation in high-fat diet-fed mice (74).

Individuals with worsening glycemic control exhibit excess AT deposition of collagens I, III, IV, and VI (59). Collagen VI gene expression coincides with more visceral adipose mass and pro-inflammatory macrophage accumulation (75, 76). Excess collagen VI deposition imparts stress by inhibiting adipocyte expansion (60). AT fibrosis creates rigidity, restrains adipocyte expansion, and, ultimately, triggers adipocyte inflammation in response to the increased mechanical stress. Collagen IV, which accounts for up to 50% of the basement membrane, also increases with TGFβ-1 and TGFβ-3 gene expression in human subcutaneous adipose, resulting in pro-inflammatory and pro-fibrotic phenotypes (77). Increases in collagens, including Col24α1, are associated with insulin resistance in AT and skeletal muscle (78, 79). Also, gene expression of CD44, which regulates cell–cell and cell–matrix interactions, is 3-fold higher in subcutaneous AT of MUO individuals, and CD44 density on macrophages is associated with the M1 phenotype (80). These data indicate that a vicious cycle of aberrant ECM turnover and increased inflammatory signaling, including M1 macrophage recruitment, results in insulin resistance in obese AT.

MHO individuals possess improved ECM turnover rates, as their more flexible ECM constitution allows for increased lipid storage. As previously mentioned, the expandability of MHO individuals' subcutaneous AT is thought to contribute to their decreased visceral AT accumulation and healthier metabolic profile (60), resulting in less cell death and decreased M1 macrophage recruitment (23). Decreased amounts of MMPs and TIMPs, including TIMP-1, allow MHO adipocytes to differentiate more readily (81), as insulin-sensitive obese patients demonstrate less fibrosis than diabetic obese patients before and after bariatric surgery (82). Improving ECM turnover in MUO individuals could permit increased subcutaneous fat mass and ameliorate metabolic dysfunction and shifts in macrophage balance (83). M2 secretion of TGF-β provides essential structural support and necessary remodeling. However, in pathological instances, this secretion results in aberrant fibrosis development. Secretion of TGF-β by M2 macrophages is intended to promote anti-inflammatory tissue remodeling, though if aberrant, results in increased collagen deposition, downstream fibrosis, and insulin resistance (84). MHO individuals' M2/M1 AT macrophage ratio, corresponding TGF-β secretion, and adequate ECM turnover allow for decreased adipocyte mechanical stress.

Genes and gene product regulation in different obesity subtypes also determine ECM turnover by controlling macrophage polarization. For example, microRNAs (miRNAs) alter gene expression and modulate downstream glucose metabolism and insulin sensitivity in obesity (85), and exosomes from AT-derived stem cells control M2 macrophage polarization (86). Specifically, exosomal miRNA-34a secreted by adipocytes suppresses M2 macrophage polarization and promotes obesity-induced adipose inflammation and metabolic dysfunction (87), whereas increased expression of miRNA-145 in visceral adipose reduces macrophage expression of pro-inflammatory cytokines through adenosine diphosphate ribosylation factor 6 (88). MiRNA-145 also promotes preadipocyte differentiation and angiogenesis, leading to healthier AT (89). Adipocyte-derived exosomes contain many miRNAs still being characterized, such as miRNA-23b, miRNA-148b, and miRNA-4429, which are expected to alter TGF-β signaling and potentially mitigate downstream fibrosis (90). For example, upregulation of miRNA-23b mitigated kidney fibrosis in leptin-deficient mice (91), suggesting that exosomal miRNAs secreted from MHO adipose likely promote both anti-inflammatory macrophage polarization and preadipocyte differentiation while mitigating fibrosis.

Current data indicate that MHO adipose corresponds with sufficient ECM deposition and breakdown coupled with balanced AT macrophage populations and anti-fibrotic/pro-adipogenic miRNA secretion. Of note, miRNA promotion of preadipocyte differentiation co-occurring with new blood vessel formation (89) highlights that adipose expansion necessitates vascularization.

## Angiogenesis

Adipose vascularity dictates tissue metabolism and insulin sensitivity. In obese states, AT expansion requires neovascularization that allows for sufficient oxygenation, nutrient delivery, and adipocyte differentiation. Adipose microvasculature plays a primary role in glucose homeostasis, as impaired tissue perfusion results in decreased glucose uptake and is a hallmark of T2D (92). Adipocyte hypertrophy that can occur within just three days of high-fat diet consumption incites signals to increase angiogenesis and alleviate hypoxia (93). Successful neovascularization and its concomitant ECM remodeling reduce hypoxia to maintain AT health (94).

MHO individuals typically accumulate subcutaneous white AT, which possesses improved angiogenic capabilities as compared with visceral adipose (95). MHO individuals maintain peripheral capillary density similar to metabolically healthy lean individuals, which allows for enhanced nutrient flow, and demonstrate improved fitness compared with MUO (96–98). Vascular endothelial growth factor (VEGF) contributes to new blood vessel formation, as it induces the growth of both preexisting and new vessels (65). Adequate AT VEGF signaling in high-fat diet-fed mice protected the animals against insulin resistance by reducing hypoxia and, in turn, increasing their M2/M1 tissue macrophage profile (99–101). Increases in capillary density coincided with improvements in metabolic function in obese rats with metabolic syndrome (98). Improved fitness achieved through aerobic training of high-fat diet-fed rats increased AT capillary density and increased the number of M2 tissue macrophages (102).

Improved vascularity in MHO AT also is likely to increase numbers of adipose progenitor cells, as such cells reside within adipose vasculature (103). Increased numbers of adipose progenitors allow for hyperplastic expansion, which perpetuates an anti-inflammatory immune profile, including an increased M2/M1 macrophage ratio, as demonstrated by increased adipocyte hyperplasia in the subcutaneous AT of obese women (104). These data suggest that hyperplastic subcutaneous depot expansion that co-occurs with increased AT vascularity facilitates an anti-inflammatory AT milieu in the MHO.

Prolonged hyperglycemia makes the cells within AT vessels susceptible to injury and promotes microvascular dysfunction. A vicious cycle occurs in MUO patients, where impaired glycemic control worsens vascular reactivity, which then exacerbates AT hypoxia, inflammation, and tissue insulin resistance. MUO patients have a 44% decrease in capillary density and 58% lower VEGF signaling in the subcutaneous adipose, highlighting the occurrence of vascular rarefaction with hyperglycemia (105). Likewise, VEGF expression in both subcutaneous and visceral adipose decreases in a stepwise fashion with worsening insulin resistance (106). ECM dysregulation contributes to insufficient vascularization, as obese patients with T2D demonstrate increased basement membrane thickness (59) and higher collagen VI synthesis, which correlated inversely with AT oxygenation in MUO patients (105). The two-hit process of impaired ECM remodeling and poor vascularization stimulates a pro-inflammatory immune cell response in MUO adipose, exemplified by CD68 mRNA and macrophage inflammatory protein 1α expression inversely correlating with AT oxygenation in MUO subjects (105). Accordingly, contrary to MHO adipose, decreased angiogenic capacity and increased vessel injury in MUO adipose result in increased pro-inflammatory M1 macrophage recruitment and pro-inflammatory signaling.

As angiogenic capacity influences adipose macrophage subtypes, existing macrophages in adipose impact angiogenesis. Macrophage deletion results in a reduction of vascular density in AT (107). Macrophages are a significant source of AT PDGF, which assists with blood vessel growth and repair of damaged vessels; their deletion also leads to a significant reduction in PDGF mRNA (108). M2 macrophages promote angiogenic signaling, as evidenced by increased endothelial cells and tubular structures in subcutaneous adipose post-M2 macrophage injection (109). M2 macrophage polarization, but not the M1 phenotype, caused a substantial downregulation of TIMP-1 expression, resulting in the production of the angiogenic activated zymogen, proMMP-9 (110). These data suggest that the anti-inflammatory MHO adipose macrophage profile stimulates angiogenesis, which not only ensures sufficient AT nutrient supply but also contributes to MHO individuals' improved fitness and systemic health.

## Nutrition and Adipose Macrophage Programming

Nutrient overload caused by excess food consumption is the most common initial trigger for obesity, and diet represents the paramount environmental health factor that is both modifiable and variable across populations. High caloric intake *per se* can effect macrophage polarization (27); however, dietary factors can influence either augmentation of M1 subtype abundance or support M2 programming. Macrophages have evolved to be effective responders to pathogen-associated molecular patterns (PAMPs) and damage-associated molecular patterns (DAMPs), which share common effector pathways. These patterns have been best explored with “microbial” inputs where these pathogen signals (endotoxin, lipoproteins, membrane proteins, peptidoglycans, DNA fragments, and lipoteichoic acid are examples) bind to receptors and initiate both phagocytosis/destruction and activation of inflammatory outcomes crucial to effective innate immunity (111). Toll-like receptors (TLRs), scavenger receptors, and mannose receptors are pattern recognition receptors present in all macrophages, and binding and activation of the inflammasome and subsequent release of cytokines and interferons lead to polarization toward the M1 type of local resident macrophages, recruitment of circulating monocytes for activation, and proliferation of macrophages *in situ* (112).

Diet has profound effects on the magnitude of DAMPs and PAMPs to which adipose macrophages are exposed. One of the important observations made in people was coined “metabolic endotoxemia,” whereby caloric excess was related to increased biomarkers of microbial translocation, such as lipopolysaccharide-binding protein 1 (LBP1) (113). LBP1 is released from the liver into the circulation in response to PAMPs and functions as a co-receptor for TLRs present on the macrophage cell membrane, thus initiating inflammatory responses in peripheral tissues, such as adipose (114, 115). In addition to just caloric excess, specific dietary components are known to induce LBP1 and PAMP signaling through modulation of the microbial interactions at the intestinal mucosal barrier. Fructose is the best described dietary factor proven to increase intestinal barrier dysfunction in rodents, humans, and non-human primates (116–120). Fructose is additionally associated with excess caloric consumption, obesity, and metabolic diseases, such as diabetes and metabolic-associated fatty liver, and these disease states further augment macrophage accumulation and the inflammatory cycle of insulin resistance in AT (27). Lipoproteins are additionally recognized as PAMPs, and low-density lipoproteins reliably increase in concentration in response to caloric excess and fructose exposure, as the liver packages and processes TGs for export (121). This lipoprotein-delivered TG is the substrate for AT to uptake and store peripherally, which, in unhealthy obesity, may not be an efficient process. Impaired insulin sensitivity and excess adipocyte hypertrophy lead to hypoxia, further inflammatory signaling and macrophage recruitment, and even adipocyte apoptosis or necrosis—the sequelae being more local DAMPs to drive local macrophages to respond, recruit, and additionally augment the M1 response.

The endotoxemia resulting from caloric excess or dietary fructose has been described as sterile; however, more recently, antibiotic and probiotic therapy deployed to modify the pathogen response has shown effectiveness in improving inflammatory and metabolic outcomes (117). More evidence to suggest that local adipose PAMP responses are to actual pathogens includes the recent demonstration of an adipose microbiome in obese people (122–124). These bacteria are confirmed to include whole live organisms and be present in the circulation and visceral and subcutaneous AT depots (124). From the data suggesting that dietary calories and ingredients increase microbial translocation and shape the microbiome, it is likely that the number and type of microbes filtered out into adipose also are diet driven (125) and will influence the abundance and polarization of adipose macrophages.

Saturated fat is another dietary component with the ability to function as a PAMP/DAMP. Structurally, a longer chain of single carbon–carbon bonds may mimic the saturated fatty acids in phospholipids of most microbial membranes and the long fatty acid chains incorporated in the structure of endotoxins, which in intact gram-negative microbes reside in the outer membrane (126). Saturated fat intake has been related to endotoxemia, but studies that include calorie control are not available, and caloric excess alone is sufficient to elevate LBP1 and induce peripheral inflammation (127–129). Similarly, trans-fatty acids structurally resemble saturated fatty acids and are presumed to act as pro-inflammatory danger signals and a potent dietary ingredients famous for induction of metabolically unhealthy obesity (130, 131). A diet rich in unhealthy attributes, such as excess caloric amounts, high fructose or added sugars, cholesterol (132), saturated and/or trans-fatty acids, all drive macrophage activation through highly conserved pathways evolved to detect pathogens and resolve tissue damage (133). The result perpetuates an inflammatory state and M1 phenotypic predominance in AT in response to signals that indicate the need for active phagocytic and antigen presentation functions. Depot differences are not well-described; however, some evidence for dietary factors inducing intra-abdominal fat shifts do exist. Examples include trans-fat consumption being linked to visceral fat accumulation, and in an obese patient cohort, the abundance of ectopic bacteria in omental fat tissue was slightly higher than that in subcutaneous fat, both of which are consistent with the body of knowledge that indicates intra-abdominal AT is more contributory to unhealthy obesity than is subcutaneous fat expansion (122, 124).

Few dietary factors directly influence macrophages positively to effect an M2 inflammation-resolving state. Polyunsaturated fatty acids (PUFAs) do have a direct role on macrophage function (134), whereas most dietary components have indirect contributions to adipose health and consequential reductions in DAMP/PAMP sensing by local macrophage populations (135). These indirect effects will not be discussed, but examples include dietary fiber, which shifts the microbiome and improves mucosal barrier function, thus decreasing LBP1 and endotoxemia (136–138), dietary components, such as isoflavones, which are rich in fermented foods, and polyphenols, which are rich in fruits and vegetables. Isoflavones can have a lipid-lowering effect (139), thus decreasing lipoprotein sensing by scavenger receptors, and can have estrogen receptor (ER) activity, which indirectly can reduce inflammation and promote vascular reactivity. Macrophages express ER (predominantly α-isoforms and G-protein coupled ER1), and dietary isoflavones can bind and decrease Nuclear factor-kappa B (NF-κB) activation and cholesterol oxidation in the context of lipid and cholesterol exposure (140), thus facilitating or maintaining M2 polarization in culture and vascular tissue, an effect likely to be also seen in adipose macrophages (141). Polyphenols can be effective free radical scavengers, thus reducing local inflammation and tissue damage signaling (142, 143).

Omega-3 polyunsaturated fatty acids (n-3 PUFA) cannot be synthesized *de novo* by humans due to the lack of delta-12 and delta-15 desaturase enzymes and must, therefore, be acquired from the diet (144). The major n-3 fatty acid in the diet, α-linolenic acid (18:3n-3), can be converted to other more anti-inflammatory lipids, such as eicosapentaenoic acid (20:5n-3), docosahexaenoic acid (22:6n-3), and the less recognized docosapentaenoic acid (22:5n-3), which can be directly sourced through consumption of fish and derived fish oils. The utilization of dietary n-3 fatty acids in the synthesis of complex PUFAs, such as docosahexaenoic acid, eicosapentaenoic acid, and anti-inflammatory prostaglandins is well-noted and thought to contribute to the reduction of pathologies associated with chronic disease, including metabolic syndrome. The challenge is that the conversion of α-linolenic acid into these anti-inflammatory lipids is very limited in people; thus, increasing dietary intake, coupled with counseling to reduce the negative dietary features described earlier, is a popular strategy to improve metabolic health in obesity. N-3 PUFAs directly interact with G-protein coupled receptor (GPR) 120 to generate an intracellular signaling complex that inhibits multiple inflammatory pathways, such as NF-κB and activated c-Jun N-terminal kinase, which are downstream of TLR and cytokine receptors (145). This effect is not limited to macrophage signaling and shifting the profile toward a resolving M2 phenotype; these healthy long-chain fatty acids also signal through GPR120 on adipocytes to reduce inflammation and improve insulin sensitivity, leading to less DAMP signaling from hypoxic, stressed adipocytes and decreased paracrine inflammatory effects on tissue-resident macrophages. In summary, healthy dietary features, such as fiber, n-3 PUFAs, and bioactive flavonoids can directly and indirectly drive the macrophage profile toward an M2 anti-inflammatory profile and a healthier state, even if the subject is obese (27). Diet can influence the balance of M1 and M2 macrophages in adipose both directly, by modifying the burden of DAMP/PAMP signaling and indirectly by influencing insulin sensitivity and tissue function of adipocytes and vascular cells in adipose. Therapeutic strategies that capitalize on dietary mechanisms are in development, including synthetic GPR120 ligands, probiotics, and synbiotics to improve intestinal barrier function, as methods to improve health in obese persons (70, 138).

## Impacts of Non-alcoholic Fatty Liver Disease on Adipose Macrophage Types

MHO individuals' liver composition and inflammatory signaling moderate the anti-inflammatory profile of their peripheral tissues. Obesity-related nutrient overload incites spillover of free fatty acids from AT that are taken up by the liver through the portal vein. Accordingly, increased visceral adipose accumulation corresponds with liver triglyceride accumulation. The severity of non-alcoholic fatty liver disease and non-alcoholic steatohepatitis has been shown to correspond with an expression of inflammatory genes in AT (146). Increased pro- and anti-inflammatory macrophage infiltration in visceral adipose was observed in obese patients with non-alcoholic steatohepatitis (146). MHO individuals demonstrate less liver triglyceride accumulation and liver fibrosis and overall improved liver function compared with MUO individuals (147, 148). Decreased liver fibrosis corresponded with fewer omental AT macrophages in obese humans, as macrophage accumulation decreased with decreasing fibro-inflammation indexes (149). Adiponectin, an adipokine that promotes AT lipid storage, lipid oxidation, and downstream anti-inflammatory signaling, is increased in MHO compared with MUO, providing another physiologic mechanism for MHO individuals' decreased liver triglyceride accumulation (150–152). Adiponectin has also been shown to correlate with insulin resistance in obese female people (153). Decreased liver triglyceride accumulation and fibrosis, along with increased effectiveness of anti-inflammatory signaling from the liver, correspond with reduced macrophage accumulation in MHO and allow for improved adipose storage and sustained AT glucose uptake.

## Paracrine Adipose Effects on the Cardiovascular System

In addition to their decreased risk of all-cause mortality, MHO individuals experience a decreased risk of heart failure even compared with metabolically unhealthy lean individuals (154). The interactions between MHO individuals' AT and their cardiovascular system explain the observed cardiometabolic outcomes.

The AT perivascular and epicardial fat depots are in direct proximity to cardiovascular tissue and interact positively in a paracrine fashion with the myocardium and vasculature in MHO persons. Perivascular AT, located around the large arteries, produces nitric oxide and secretes adipocyte-derived relaxing factors and other adipokines that relax vascular smooth muscle cells and are able to go into microcirculation (155). As perivascular adipose maintains vascular bed homeostasis, it controls the effects of insulin on microcirculatory systems in metabolic tissues. For instance, perivascular adipose successfully facilitates insulin-mediated vasoreactivity and glucose uptake in skeletal muscle (156). Interestingly, loss of perivascular AT in lipoatrophic mice (A-ZIP/F1) enhances the contractile responses of blood vessels, which results in hypertension (157). In instances of pathological perivascular dysfunction, the perivascular AT release of adipocyte-derived relaxing factors diminishes, whereas its release of pro-inflammatory cytokines, including IL-6, tumor necrosis factor-alpha, and MCP-1, increases, and a negative cycle of perfusion and AT dysfunction perpetuates as described earlier. This directly impacts endothelial and vascular smooth muscle cells and incites vascular inflammation (155). Pro-inflammatory perivascular AT signaling is initiated by decreased nitric oxide production, increased reactive oxygen species, and pro-inflammatory cytokines released by the dysfunctional endothelium, vascular smooth muscle cells, or vascular macrophages (155). In MUO individuals, dysfunctional perivascular AT alterations stem from adipocyte hypertrophy, hypoxia, oxidative stress, and pro-inflammatory macrophage infiltration (158, 159). These data suggest that MHO perivascular AT successfully facilitates glucose uptake while promoting anti-inflammatory macrophage accumulation.

Like perivascular AT, interactions between healthy epicardial AT and the myocardium mitigate pro-inflammatory signaling in MHO individuals. Epicardial AT, located between the myocardium and visceral pericardium, acts as an energy source for the myocardium, as epicardial adipose has a higher capacity for uptake and release of free fatty acids and a lower rate of glucose utilization than other visceral depots (160). Given its ability to take up free fatty acids, epicardial adipose is thought to act as a buffer for the myocardium against lipotoxicity (160, 161). However, in pathological settings, the epicardial adipose may provide excess free fatty acids associated with myocardial steatosis and systemic insulin resistance (162). Insulin resistance and T2D are associated with increased MCP-1 expression in epicardial adipose, and peri-coronary adipose displays increased M1 macrophage infiltration compared with other regions distal to the coronaries (163, 164). The importance of whole-body health highlights that peri-coronary epicardial adipose inflammation may influence vascular function negatively as well as positively (163). In unhealthy obese states, hypoxic perivascular adipose transports macrophages that may carry oxidized cholesterol from systemic circulation to epicardial adipose through the neovascularized vasa vasorum (165, 166). Local epicardial AT inflammation also stems from dysregulated miRNA expression. Patients with coronary artery disease demonstrate increased miR-103-3b upregulation, which is a potential modulator of the pro-inflammatory cytokine CCL13 (167). Insulin resistance and T2D are characterized by changes in miRNAs, including miR-29a and miR-143, which regulate AT browning and inflammation (168). Importantly, miR-29a has been associated with myocardial fibrosis, whereas miR-143 is a biomarker of vascular smooth muscle cell activation that is linked to atherosclerosis and hypertension (169, 170). Adipose acts as a local renin–angiotensin system by producing angiotensinogen, a precursor to angiotensin II (171). The hypertension medication telmisartan—an angiotensin II type 1 receptor blocker and PPARγ agonist—improved insulin resistance while decreasing M1 and increasing M2 macrophage gene expressions in visceral adipose from high-fat diet-fed mice (171). These data suggest that a hypertensive MUO person who has more M1 macrophages recruited into their adipose, when treated with telmisartan, may experience a shift in their adipose macrophage profile and a reduction in local inflammation. While epicardial AT function protects MHO individuals from lipotoxicity and maintains the anti-inflammatory immune cell profile, more work is needed to understand the crosstalk between epicardial adipose miRNAs, the cardiovascular system, and their relationship to health and disease.

## Discussion

In this review, we discuss how macrophage phenotypes drive adipose health in MHO and MUO persons, as these immune cells affect the local AT niche and are heavily influenced by diet and systemic health characteristics (Figure 1). Improved white adipose function in conjunction with the consumption of n-3 PUFAs, polyphenols, and fiber results in anti-inflammatory M2 macrophage programming in MHO. Functional white adipose adipogenesis, increased tissue vascularization, ECM turnover, and downstream anti-inflammatory signaling in combination with consumption of the dietary components mentioned earlier propagate M2 maintenance and proliferation while abating harmful pro-inflammatory M1 macrophage recruitment. Gut mucosal barrier integrity, functional liver-adipose, and cardiovascular system-adipose cross talk parallel the effects of diet by decreasing MHO AT pro-inflammatory signaling and maintaining insulin sensitivity. Alternatively, increased dietary consumption of saturated fat, cholesterols, *trans* fat, and fructose incites pro-inflammatory adipose macrophage recruitment in MUO adipose, which inhibits adipogenesis. Consumption of these dietary components, in conjunction with dysfunctional adipogenesis, results in augmented adipocyte hypertrophy. This, combined with decreased angiogenic signals, disrupted ECM turnover, and downstream pro-inflammatory cytokine secretion, stimulates further pro-inflammatory M1 macrophage recruitment. Impaired gut mucosal barrier integrity in the MUO drives multi-organ inflammation. This results in dysfunctional adipose-liver and adipose-cardiovascular system cross talk, which concurrently promote pro-inflammatory M1 macrophage recruitment in MUO adipose. Once M1 macrophages enter the tissue, they secrete additional pro-inflammatory cytokines that recruit more M1 macrophages. This vicious cycle of inflammation and perpetuation of unhealthy AT, further expansion, and greater multisystemic dysfunction characterizes MUO individuals.

Clearing M1 macrophages from unhealthy adipose may reestablish metabolic health. Obesity incites senescent cell accumulation in AT and M1 macrophage recruitment, and pharmacological senescent cell clearing agents have effectively reduced macrophage accumulation and pro-inflammatory signaling while restoring metabolic function in obese mice and people (172–174). Calcium/calmodulin-dependent protein kinases (CaMKs) play roles in myocardial ischemia/reperfusion injury, regulating food intake and energy expenditure. Activation of CaMK II δ in cardiomyocytes prompted pro-inflammatory macrophage recruitment and associated NF-κB signaling that results in fibrosis and myocardial dysfunction (175). Loss of CaMK kinase II (CaMKK2) reduced AT M1 macrophage-derived NF-κB signaling caused by a high-fat diet, highlighting an important function for CaMKK2 in controlling diet-induced adipose M1 macrophage inflammation (176). For example, treatment with a CAMKK2 inhibitor, tilianin, decreased pro-inflammatory signaling in cardiomyocytes (177). The use of CaMKK2 inhibitors in MUO may reduce AT inflammation, although more research is needed to determine how CaMK inhibition impacts AT immune cell populations over time.

As obesity rates continue to rise and weight-loss interventions prove largely unsuccessful, understanding how to mediate the vicious AT macrophage cycle in MUO individuals is imperative. Although long-term obesity ultimately increases the risks of multisystem adverse events, breaking the pro-inflammatory macrophage cycle will potentially shift MUO individuals to MHO and reduce current health burdens.

## Author Contributions

All authors listed have made a substantial, direct and intellectual contribution to the work, and approved it for publication.

## Conflict of Interest

The authors declare that the research was conducted in the absence of any commercial or financial relationships that could be construed as a potential conflict of interest.
